# Can remote assistance for robotic surgery improve surgical performance in simulation training? A prospective clinical trial of urology residents using a simulator in south america

**DOI:** 10.1590/S1677-5538.IBJU.2022.0104

**Published:** 2022-08-28

**Authors:** Arie Carneiro, Oliver Rojas Claros, Jonathan Doyun Cha, Paulo Priante Kayano, Marcelo Apezzato, Andrew Aurel Wagner, Gustavo Caserta Lemos

**Affiliations:** 1 Hospital Israelita Albert Einstein Departamento de Urologia São Paulo SP Brasil Departamento de Urologia, Hospital Israelita Albert Einstein, São Paulo, SP, Brasil; 2 Beth Israel Deaconess Medical Center and Harvard Medical School Boston MA USA Beth Israel Deaconess Medical Center and Harvard Medical School, Boston, MA, USA

**Keywords:** Robotic Surgical Procedures, Computer Simulation, Clinical Trials as Topic

## Abstract

**Introduction:**

We aimed to evaluate the role of remote proctoring during the initial training phases of a robotics curriculum using surgical robot skills simulator exercises.

**Materials and Methods:**

Prospective randomized study comprising 36 urology residents and junior staff urologists without previous robotic training. Group 1 (G1) performed exercises without any assistance or support, group 2 (G2) received support from in-person proctor, and group 3 (G3) from a remote proctor through a telementoring system. Qualitative and quantitative analyses were conducted for each exercise and group.

**Results:**

The overall score approval rates (OSA) for the different skill exercises were Ring Walk 2 (RW2) 83%, Energy Dissection 2 (ED2) 81%, and Ring Walk 3 (RW3) 14%. RW2 OSA was higher on attempt 3 than on attempt 1 (83.3% vs. 63.9%, p=0.032). ED2 OSA rate was higher in attempt 3 than in attempt 1 (80.6% vs. 52.8%, p=0.002). RW2 OSA was similar among the groups. In ED2, both remote and live assistance were significantly related to upper OSA (G1=47.2%, G2=75.0%, G3=83.3%, p=0.002). RW3 had similar OSA among the groups, which can be explained by the high level of difficulty and low OSA in all the groups. However, in a sensitive quantitative analysis, the mean overall score of the participants in RW3 was higher in both proctored groups (G1=24, G2=57.5, G3=51.5, p=0.042).

**Conclusion:**

Robotic performance increased significantly over three attempts for simulation exercises of low, medium, but not high-complexity. Proctoring, either in-person or remotely, has a positive impact on approval performance, particularly in intermediate tasks.

## INTRODUCTION

Robotic technology has been used during surgery for approximately 30 years. However, its use during urologic surgery has grown significantly over the last decade due to improvements in visualization and precision compared to standard laparoscopy (1).

Robotic training programs in developing countries differ from those in the United States and Europe. For example, there are few or no uniform or well-established resident or fellowship training programs for young surgeons interested in robotics. Busato et al. demonstrated that robotic surgery is still largely uncharted territory for far from Brazilian residents (2). Meanwhile, in other countries, robotic knowledge is currently at urology objective structured clinical examinations (OSCEs) stages (3). Surgical education has recently undergone a paradigm shift towards competency-based frameworks, highlighting the need for surgical training, evaluation, and certification (4).

In developing countries, higher-volume robotic centers are mainly concentrated in large cities, forcing most expert surgeons to travel for proctoring, which is a strain on already limited time and resources and, even worse, this limits the dissemination and development of robotic surgery in these countries (5, 6).

To reduce inequalities in health resources in Brazil, programs such as the Institutional Development Program of the Unified Health System (*Programa de Desenvolvimento Institucional do Sistema Único de Saúde – PROADI-SUS*) have been developed since 2008. Among these programs, telemedicine stands out, in which high-tech hospitals remotely connect with health units to support them through the exchange of knowledge, information, and experiences. De Souza et al. demonstrated that telemedicine is an excellent tool for disseminating knowledge about these services (7). As our service is part of the PROADI-SUS project from an important robotic surgery center, we saw the opportunity to combine these fronts.

Inanimate and virtual reality simulations have played a significant role in robotic surgery training, and studies have shown that basic robotic skills can be transferred from simulators to the operating room (3, 8). The da Vinci Surgical Skills Simulator (dVSSS) of the da Vinci robotic surgical system™ (Intuitive Surgical, Inc.) is a validated virtual training system that allows repetitive skill training at different pre-determinate proficiency levels. It provides trainees with timed scores (9), which can help enhance the learning curve.

A user-friendly telementoring platform may play an essential role as a tool to share expertise and spread knowledge (10), allowing larger centers to assist smaller ones in improving safety and shortening the learning curve. Our institution currently has an established program of telementoring in emergency and critical care medicine, and we sought to build on that expertise.

The telementoring platform we describe herein could provide a final “testing” for the surgical trainee prior to embarking on actual live surgery. Moreover, it can be used as a means to share experiences in complex and rare cases.

We hypothesized that remote guidance using the dVSSS exercises would positively impact the performance score required by urology residents and young urologists. We aimed to validate a telementoring system using the dVSSS with the specific goal of hastening the training process with simulators for novice and intermediate surgeons.

### Objective

This study aimed to evaluate the role of remote proctoring during the initial training phases of a robotic curriculum using dVSSS exercises performed by urological residents and junior attending-level urologists.

## MATERIAL AND METHODS

A prospective, randomized study of remote proctoring in simulation training was conducted at the *Hospital Israelita Albert Einstein*, São Paulo, SP, Brazil, from March 2016 to August 2017. The study was approved by the Research Ethics Committee of the *Hospital Israelita Albert Einstein*(IRB number 62289516.0.0000.0071). Each participant enrolled in this study signed an informed consent form for the survey and agreed to the use of the related data for research purposes.

The participants were urology residents and junior staff urologists who had no previous contact with the robotic platform. Thirty-six participants were included in the study; 12 in each group. Of the participants, 18 were residents of the first and second years of medical residency in urology, and 18 were residents of the third year of medical residency in urology or junior staff urologists. Two surgeons were excluded from the study because of previous contact with the robotic platform. Only one proctor was included in the study who participated as an in-person proctor (Group 2) and telementoring proctor (Group 3).

All participants enrolled in the study received a standardized oral orientation and demonstration of the management and use of the robotic platforms, with details on the correct management of the console and simulator. Subsequently, participants completed an initial basic training protocol consisting of five exercises in the dVSSS (Camera Targeting 1, Camera Targeting 2, Ring Walk 1, Energy dissection 1 and Play Ground). Each exercise was performed thrice (11). To set the exercises and number of repetitions, a pilot study was conducted with five urology residents without any familiarity with the robotic platform, and three experts. Based on the results of this study, we selected the exercises and number of repetitions in consensus among the authors. We determined the difficulty level, exercises, and repetitions for this study based on the literature and our experience (9).

The participants underwent simple randomization by lottery into three groups. Group 1 (G1) performed the exercises without assistance or support. Group 2 (G2) had support from an in-person proctor. Group 3 (G3) was supported by a remote proctor through a telementoring system, as described below (AdobeConnect®).

Only one proctor was proficient in performing dVSSS exercises and was consistent throughout the training exercises. He only provided oral advice.

After randomization, three exercises were performed three times each to evaluate skill progression. These were the low-difficulty Ring Walk 2 (RW2), intermediate-difficulty Energy Dissection (ED2), and high-difficulty Ring Walk 3 (RW3).

### Outcomes and Performance evaluation

Qualitative (the participant reaches proficiency or not, “overall score approval rate”) and quantitative (overall score: 0–100 points) proficiency evaluations were performed for each attempted exercise using the dVSSS scoring system. In addition, the time taken to complete the exercise, economy of motion, instrument collisions, excessive force applied to an instrument, instruments out of view, master workspace range, and drops were recorded and analyzed.

### Telementoring system

The telemonitoring system consisted of two stations: a compatible computer and high-quality internet connection for the remote proctor, and a telemonitoring cart connected to the robot for the surgeon. The image from the robot went to the cart, which was connected via AdobeConnect®. Through the internet, the proctor could access this image and provide remote support, which consisted of the same oral advice as in G2.

AdobeConnect® was the system used for communication between the research candidate and the remote proctor. It provides a very good image quality with a short delay (1 s). This system is commonly used in Brazil and elsewhere for teaching conferences, and an additional advantage is that only one center is required to own the software.

Basic IT hardware was employed: a PC running Windows 7 (Intel i3, 4 GB RAM, HD 80 GB), graphics board set at 1280 × 1024 resolution, network card with a minimum connection speed of 100 Mbps, Internet Explorer version 9.0, monitor with a resolution of 1280 × 1024, and audio system with a microphone and headset.

#### Statistical Analysis

We used the chi-square test to compare the groups regarding participants’ degree of training. To investigate the effects of the attempt, group, and degree of training, we used generalized estimation equations. We compared the performance in the different groups separately, using the Friedman and Kruskal–Wallis tests. In cases of significant differences, multiple comparisons were performed using the Bonferroni's method. The analyses were carried out on SPSS, version 24.

## RESULTS

Table-1 summarizes the data of the study participants. The participants were predominantly men, and only one participant had a left-predominant hand. The groups studied had similar levels of training (p=0.717, chi-square test), number of surgeries performed per month (p=0.913, Fisher's exact test), and age (p=0.986, variance analysis).

**Table 1:** General characteristics of the participants according to groups.

Variables		Group 1 (no proctor)	Group 2 (in person proctor)	Group 3 (telementoring proctor)
**N**		12	12	12
**Gender**				
	Male	11 (91.7%)	12 (100.0%)	8 (66.7%)
	Female	1 (8.3%)	0 (0.0%)	4 (33.3%)
**Dominant hand**				
	Right	11 (91.7%)	12 (100.0%)	12 (100.0%)
	Left	1 (8.3%)	0 (0.0%)	0 (0.0%)
**Degree of training 1**				
	R3	0 (0.0%)	5 (41.7%)	2 (16.7%)
	R4	6 (50.0%)	2 (16.7%)	3 (25.0%)
	R5	3 (25.0%)	3 (25.0%)	6 (50.0%)
	Urologist	3 (25.0%)	2 (16.7%)	1 (8.3%)
**Degree of training 2**				
	R3+R4	6 (50.0%)	7 (58.3%)	5 (41.7%)
	R5 + Urologist	6 (50.0%)	5 (41.7%)	7 (58.3%)
**Numbers of surgeries performed per month**				
	≤ 4	3 (25.0%)	6 (50.0%)	6 (50.0%)
	≥ 5 or ≤ 7	5 (41.7%)	3 (25.0%)	2 (16.7%)
	> 7	4 (33.3%)	3 (25.0%)	4 (33.3%)
**Age**				
Mean (SD)		31 (2)	31 (4)	31 (2)
	Min-Max	28 - 37	26 - 40	28 - 36

**R3** = First year of medical residency in Urology; **R4** = second year of medical residency in Urology; **R5** = Third year of medical resindecy in Urology.

The most common corrections were to improve the use of the Endowrist manipulation, improve the use of both hands, keep the instruments close to each other, avoid leaving the camera too close, and avoid keeping the instruments out of view.

We observed differences in difficulty between the exercises. We observed differences in difficulty between the exercises. Considering the attempts, the first attempt showed RW2=64%, ED2=53% and RW3=8%; the second attempt showed RW2=69%, ED2=72% and RW3=11%; and the third attempt showed RW2=83%, ED2=81% and RW3=14%; respectively. Based on the overall score approval rate (OSA), RW3 provided a significantly greater degree of difficulty than other exercises on the third attempt (OSAs: RW2= 83%, ED2=81%, RW3=14%) (Appendix: Supplementary Table-1).

We also observed different levels of performance regarding the number of attempts. The OSA increased significantly with the number of attempts only in RW2 and ED2. The RW2 OSA was higher on attempt 3 than on attempt 1 (83.3% vs. 63.9%, p=0.032). The ED2 overall score approval rate was higher in attempt 2 than in attempt 1 (72.2% vs. 52.8%, p=0.048) and in attempt 3 than in attempt 1 (80.6% vs. 52.8%, p=0.002). There was no significant difference in the OSA with respect to different attempts at RW3 (Table-2).

**Table 2:** Overall score for all participants along multiple attempts in each exercice.

Overall score	Attempt			p-value	
	1^st^	2^nd^	3^rd^	1^st^ × 2^nd^	1^st^ × 3^rd^
RW2 approval	23 (63.9%)	25 (69.4%)	30 (83.3%)	0.528	0.032
ED2 approval	19 (52.8%)	26 (72.2%)	29 (80.6%)	0.048	0.002
RW3 approval	3 (8.3%)	4 (11.1%)	5 (13.9%)	0.705	0.417

ED2 = Energy Dissection 2; RW2 = Ring Walk 2; RW3 = Ring Walk 3

We found different performance levels between the groups in the analysis of telementoring impact (Table 3). On ED2, both remote and live assistance were significantly related to upper OSA (G1 = 47.2%, G2=75.0%, G3=83.3%, p=0.002). The RW2 and RW3OSAs were similar among the groups. However, in a sensitive quantitative analysis, the mean overall score of the participants in RW3 was higher in both proctored groups (G1=24%, G2=57,5%, G3=51,5%, p=0,042).

**Table 3:** Impact of the Telementoring.

Overall Score Approval	Groups			p-value	
	G1	G2	G3	G1 vs. G2	G1 vs. G3
RW2	26 (72.2%)	27 (75.0%)	25 (69.4%)	0.769	0.703
ED2	17 (47.2%)	27 (75.0%)	30 (83.3%)	0,005	0.002
RW3	3 (8.3%)	1 (2.8%)	8 (22.2%)	0.387	0.086

ED2: Energy Dissection 2; RW2: Ring Walk 2; RW3: Ring Walk 3.

## DISCUSSION

Significant technological progress has been achieved in minimally invasive surgery using the Da Vinci® robot, and robotic surgery is successfully and reliably applied in the treatment of urological cancers (4, 11–13). Robotic prostatectomies have been introduced with the expectation of minimizing perioperative and postoperative complications, providing three-dimensional magnification and tools with seven degrees of freedom that can duplicate hand movements. However, the absence of tactile feedback and high costs are disadvantages that still need to be overcome (11).

In developing countries, this robotic platform is restricted to a few large centers (5), and the system costs and time required for robotic surgery training are major challenges that likely limit the expansion of robotic surgery (13). Telemedicine has been used to overcome distance barriers and improve access to medical services that are not consistently available in distant communities (7).

This study aimed to evaluate the role of remote proctoring during the initial training phases of a robotic curriculum using dVSSS exercises performed by urology residents and junior attending-level urologists. We observed that telementoring for novice robotic surgeons, either in-person or remotely, positively impacted performance score required by, particularly when learning intermediate simulation tasks. In ED2, both remote and live assistances resulted in significantly upper OSA (G1=47.2%, G2=75.0%, G3=83.3%; p=0.002).

Proctoring (in-person or remote) had no impact on high- and low-difficulty tasks. The RW2 OSA was similar between groups. RW3 had similar OSA among the groups, which can be explained by the high level of difficulty and low OSA in all groups, suggesting that telementoring may be helpful for beginner surgeons in allowing for more rapid attainment of higher skill levels.

We believe that by using telemedicine equipment and platforms as a means to spread knowledge, telementoring may play an essential role in the vital early step of the learning process (14). This early step is one where novice surgeons develop habits, whether helpful or harmful, that can persist throughout their learning process and even, conceivably, throughout their careers. Our study suggests that this system can augment the positive effects of simulators on the learning curve (9).

A recent review of telementoring and telesurgery categorized 38 studies into four advancing levels: verbal guidance, guidance with telestration (indicating target areas on the local monitor screen), guidance with tele-assisted surgery (controlling the operative camera or an instrument via robotic arms), and telesurgery (performing surgery remotely). Eight studies on telementoring with verbal guidance were included which reported the following related advantages: low-cost, widely available equipment, mature technology, and lower network bandwidth requirements (15). This is the first prospective and randomized study to evaluate telementoring with verbal guidance in robotic surgery training using objective dVSSS data. Additionally, we found that it is possible to build a useful mentoring system using basic infrastructure and widely available Internet services.

One prospective but not randomized trial of endovascular surgery evaluated verbal guidance (16), and other studies involved a series of patients who underwent laparoscopic and robotic procedures (17–22). Our easy-to-reproduce telementoring system allowed us to demonstrate that simple instruction from a mentor in the form of verbal guidance can be effective in teaching surgical skills remotely. Hung et al. concluded that safety, legal, financial, economic, and ethical concerns persist, for more advanced interactions (through surgical telementoring and telesurgery) to be fully adopted and clinically integrated (16).

Despite the efforts of numerous organizations, a consensus for training, credentialing, and assessment of competency in robot-assisted urologic surgery has not yet been achieved, even in developed countries (23). One of the main barriers to expanding robotic expertise in Brazil is because of its limited number of surgical simulators and proctors. Most of the 60 da Vinci robots in Brazil are concentrated in the São Paulo area. Moreover, proctors must travel to assist surgeons throughout the country; this implies a significant strain on resources.

The limitations of our study include the small number of participants in each group and the number of exercises performed. Additional information could have been obtained by adding cameras showing the actual movements of the trainees at the console, and/or recording the actual advice given by the proctor. Moreover, the participation of only one proctor can have influence in the results, by some inherent biases from this professional, such as bias in surgical practice, teaching and communication skills, due to proctor medical training, and others. A larger number of students and proctors are recommended in future studies.

Telementoring assistance can be used to provide support and increase surgeon confidence during either primary proctoring or follow-ups (24). This might allow for greater access to the limited number of trained robotic surgeons and improvements in mentoring for unusual surgeries (4).

## CONCLUSIONS

As expected, repetitive training increases the success rate of surgical robot skills. Our study showed that proctoring through telementoring, either in-person or remotely, positively impacts performance, especially with the intermediate-difficulty tasks of the dVSSS simulator. Telementoring proved to be as efficient as in -person proctoring.

## APPENDIX:

**Supplementary Table-1:** dVSSS scores.

		OSA (%) RW2			OSA (%) ED2			OSA (%) RW3		
ID	Group	First attempt	Second attempt	Third attempt	First attempt	Second attempt	Third attempt	First attempt	Second attempt	Third attempt
1	3	84	91	84	79	95	100	71	85	62
2	3	96	98	99	95	89	96	90	73	72
3	2	94	99	100	86	95	93	70	77	18
4	1	31	27	67	59	69	62	0	0	36
5	1	92	76	94	83	79	89	42	41	42
6	2	67	68	74	78	99	96	44	35	70
7	3	77	84	90	80	69	82	43	29	59
8	1	81	67	78	65	78	84	25	37	49
9	2	59	75	88	51	82	88	50	42	68
10	2	44	88	95	83	94	98	73	63	65
11	2	88	96	86	98	48	73	70	61	73
12	1	85	82	51	86	96	97	21	44	37
13	1	58	87	90	48	64	52	0	29	62
14	3	97	94	93	91	95	95	86	79	94
15	3	94	95	90	79	83	88	23	48	54
16	2	68	91	87	63	77	87	67	41	73
17	1	85	92	99	69	71	85	23	73	91
18	2	89	91	98	94	96	99	57	50	63
19	1	88	92	96	81	89	96	40	61	41
20	1	86	91	87	92	100	95	22	48	53
21	2	80	67	85	78	80	68	18	48	39
22	2	96	91	90	97	96	98	82	79	75
23	3	80	86	95	53	90	95	45	80	70
24	2	93	91	98	71	86	96	58	65	62
25	1	93	92	97	79	79	79	33	87	79
26	3	59	68	59	86	88	93	0	7	31
27	3	40	68	63	78	100	95	0	14	68
28	1	88	94	91	75	74	77	78	74	80
29	3	96	98	94	88	88	89	73	77	85
30	3	75	96	91	78	82	85	58	76	57
31	3	70	79	92	81	100	86	15	51	16
32	1	90	92	94	81	98	87	60	67	66
33	3	77	94	89	86	90	98	73	88	87
34	2	94	70	98	97	89	95	45	25	30
35	1	36	42	80	71	87	79	12	0	26
36	2	80	88	89	86	95	100	53	76	46

ID = Participant identification; Group 1 = no proctor; Group 2 = in person proctor; Group 3 = telementoring proctor; OSA = overall score approval; ED2 = Energy Dissection 2; RW2 = Ring Walk 2; RW3 = Ring Walk 3.
